# Impact of minority perceived discrimination on resistance to innovation and moderating role of psychological distress: Evidence from ethnic minority students of China

**DOI:** 10.3389/fpsyg.2022.989961

**Published:** 2022-10-04

**Authors:** Junwei Wang, Musarat Shaheen

**Affiliations:** ^1^School of Marxism, Shandong Yingcai University, Jinan, China; ^2^Bahauddin Zakariya University, Multan, Punjab, Pakistan

**Keywords:** minority perceived discrimination, resistance to innovation, psychological distress, job dissatisfaction and ethnic minority students, psychological behavior

## Abstract

Ethnic minority groups usually face discrimination in the form of prejudice and stereotypes. The self-esteem and psychological well-being of ethnic minority groups are adversely impacted by the prejudice and discrimination behavior of others. The perceived discrimination unfavorably influences the attitude and behavior of ethnic minority groups, which in turn develops resistance to innovation among them. With the support of social exchange theory, this study hypothesized that perceived discrimination positively enhances resistance to innovation and job dissatisfaction for empirical investigation. The current study also proposes that job dissatisfaction positively correlates with resistance to innovation. This study further assumes the mediating role of job dissatisfaction and moderating role of psychological distress forfurther investigation. For empirical investigation, the present study collected the data from 328 Ethnic Minority Students of various Chinese universities through a structured questionnaire method using a convenient sampling technique. This study applied partial least square structural equation modeling (PLS-SEM) for empirical examination using Smart PLS software. The findings confirm that perceived discrimination positively correlates with resistance to innovation and job dissatisfaction, respectively. It is also verified that perceived discrimination positively impacts job dissatisfaction. The results further interpreted that job dissatisfaction mediates the relationship between perceived discrimination and resistance to innovation. Additionally, the findings revealed that psychological distress does not moderate the relationship between perceived discrimination and resistance to innovation; however, psychological distress moderates the relationship between job dissatisfaction and resistance to innovation. The findings serve the organizations by pointing out the role of perceived discrimination on job dissatisfaction. This study also provides valuable theoretical and practical implications.

## Introduction

Differential treatment based on ethnicity toward a member of an ethnic group or the entire ethnic group that put them at a disadvantage can be defined as ethnic discrimination (Quillian, 1995). Ethnic discrimination occurs when people having ethnic prejudice use their power to influence the life experiences or life chances of a particular ethnic group to satisfy their prejudicial instinct (Gillborn, 2003). However, this power to influence the life experiences of a particular ethnic group is not spread across the society equally rather ethnic discrimination from some toward the ethnic group can be more than those who have less power. The impact of ethnic discrimination at school at the hands of teachers and fellow students can have a variety of negative impacts on multiple factors such as a sense of school belonging, confidence, mental well-being and academic performance (Benner et al., 2018). Discrimination based on ethnicity has drastic outcomes generally. Mental health and academic performance are worst affected. There has not been much work on the consequences of such discrimination from the perspective of “types” of perpetrators. Nevertheless, limited evidence does suggest that different perpetrators have different consequences for the victims (Benner et al., 2018). Such as the discrimination coming from the teachers themselves has shown more negative academic consequences for a victim student as compared to the discrimination coming from peers. Though, the ramification of peer-inflicted discrimination has different outcomes that entail socioemotional anxiety and distress. As far as perceived discrimination is concerned, it is the perception of an individual that is based on the sense that he/she is treated differently or unfairly merely because of his/her ethnic outlook (Cardo, 1994). Furthermore, differentiated behavior faced because of ethnicity leads to the alienation of individuals. Anger, distrust, anxiety, and lack of self-esteem are found in ethnically discriminated people. These negative behavioral traits lead to negative behaviors vis-à-vis work or study. These negative behaviors result in job dissatisfaction, resistance to innovation, and psychological distress.

Both current and previous research empirically supports these concerns shown by the experts who have worked on job dissatisfaction and resistance to innovation. The literature review has demonstrated that the students who have experienced psychological distress also show signs of job dissatisfaction accompanied by resistance to innovation (Saravanan et al., 2020). These students come from various backgrounds academically and ethically which shows that these issues are being experienced by students worldwide. The students have shown their concerns about these mental health issues while attending the university counseling centers (Andersson et al., 2021). The worldwide prevalence of job dissatisfaction and resistance to innovation among students have urged researchers to consider this subject to highlight these problems and to provide a permanent solution that can save the coming generations from falling into mental health issues (Chen et al., 2020). Increased psychological distress and mental health issues have wider implications like poor academic outcomes, poor health behaviors, and poor organizational behaviors. The literature identifies the key factors linked with the psychological distress and attributes of the students as these attributes can serve as favorable points of intervention for minimizing the risks of mental health issues and negative behaviors associated with these issues (Andersson et al., 2021).

The present study addresses the available study gaps in the literature. Ethical discrimination and resistance to innovation have long been under study. The already available literature shows the studies in which the negative effects of minority perceived discrimination inthe workplace have been revealed by the researchers (Ng et al., 2020). The current study has used psychological distress as a moderator between the perceived discrimination, resistance to innovation, and job dissatisfaction because previous research studies have shown that perceived discrimination has affected the mental health of individuals by increasing the stress and depression scores among individuals with minority status (Everett et al., 2016). According to a study perceived discrimination mediates the relationship between the minority status and psychological symptoms among the community workers (Hermanto et al., 2020). A study has explored that perceived racial discrimination increases suicidal risks. among the minority individuals (Wang et al., 2021). According to a study conducted on secondary school students, the mental well-being of the students is highly associated with their status. The students who are experiencing discrimination and belong to a perceived minority show reduced mental well-being (Kokkonen et al., 2021). Despite a lot of research being carried out in this domain, no study has investigated the impact of minority perceived discrimination on ethnic students in China. The current study aims to study the impact of minority perceived discrimination on resistance to innovation keeping in view the effects and relationships of variables like psychological distress and job dissatisfaction among ethnic Chinese students because no study has been conducted by using all these variables in a single framework. The study investigates the mediating role of psychological distress and job dissatisfaction by studying the effects and relationships of perceived discrimination, psychological distress, job dissatisfaction, and resistance to innovation. This aspect makes the current study an innovative way to fulfill the research and study gaps in this domain of research. The social exchange theory provides the theoretical underpinning for the current study.

The specific objectives of this study are as follows: Firstly, to study the impact of perceived discrimination on resistance to innovation and to explore the moderating role of psychological distress among ethnic students of China. Secondly, to explore the relationship between job dissatisfaction and resistance to innovation and to study the moderating role of job dissatisfaction between perceived discrimination and resistance to innovation. Thirdly, to verify the moderating effect of psychological distress on the relationship between perceived discrimination job dissatisfaction, and resistance to innovation. To achieve these objectives, hypothesis development has been carried out by an extensive literature review.

The current study has significant theoretical as well as practical implications. This study adds valuable literature on the subject of research, which helps in a better understanding of the subject. The practical implications of this study provide useful and practical suggestions to researchers, educationists, teachers, entrepreneurs, as well as students on ethnic discrimination and its impact on the well-being of students. The formation of this study is as follows: The introduction section presents the objectives and research purpose. The next section is a literature review and hypotheses development, which includes the theoretical underpinning and review of the available relevant literature. The next section presents the research model, statistical analysis, and result interpretation. In the end, the article presents the discussion, conclusion, limitations, and implications.

## Literature review and hypotheses development

### Theoretical support and background

Many theoretical approaches provide useful perspectives that can help explain, understand and predict the phenomenon of perceived ethnic discrimination. Social exchange theory is situated in a vast conceptual paradigm that can reach multiple social scientific realms such as anthropology, management, and psychology, etc. The name of the theory may suggest that is a unitary theory, however, as a matter of fact, it consists of many conceptual models that form a family-like structure. Both good and bad deeds are reciprocated likewise, whereas, the guiding principle in such exchanges remains dependent on the relationship between the reciprocator and the receiver (Blau, 2017). There are then different kinds of exchanges as well such as social changes as well as economic changes. Social changes are more open-ended, involve more trust, and have a flexible nature unlike economic exchanges that are mostly rigid, involve less trust, and require closer monitoring. Social exchange theory uses simple ideas and analogies like these. The framework is widely used by social scientists and researchers across multiple disciplines (Cropanzano and Mitchell, 2005). It has become a sort of research norm to use the lens of social exchange theory to probe highly pressing issues and questions regarding organizational behavior by several researchers.

Ethnic discrimination is a serious societal issue across multiple societal domains including education (D'hondt et al., 2015). Most of the available research remained particularly focused on a variety of different consequences of ethnic discrimination. However, ethnic discrimination that takes place in the context of the school environment is deeply understudied (Benner and Graham, 2013). Research in this domain can drastically enhance the likelihood of schools bringing in institutional as well as organizational changes to ensure that ethnic discrimination does not affect the mental well-being of exposed or vulnerable students in a learning environment. Furthermore, the volume of research focusing on the impact of discrimination in a multicultural education system remains even slimmer. The purpose of multicultural education systems is to provide symmetrical learning opportunities to the students irrespective of their ethnic background so that everyone can have equal opportunity and access to quality education. The studies are not only limited in number but vague as well to draw a clear picture to understand the impact of the phenomenon completely. The focus of the studies dilutes given the inclusion of varying actors such as heads of institutions, faculty, and students. Furthermore, different elements such as policies of the schools and academic culture as well as the inclusion of all age groups also negate the purpose of finding a clear pattern from the retrieved results (Apfelbaum et al., 2010). Though there is a clear relationship between discrimination and multicultural education, however, the results coming out of multiple pieces of research are contradictory given the very complex and multifaceted relationship between the two.

### Minority perceived discrimination and resistance to innovation

An act of unwillingness to adapt to change and an act of excruciating and conflicting adjustment is called resistance to innovation (Chien et al., 2022). According to social exchange theory, the principle or concept of reciprocity is mostly at work during the processes of exchanges of various natures and kinds. Resistance to innovation is a kind of behavior exchanged in an organization in response to the treatments like perceived discrimination. Minority perceived discrimination is a state of mind where a person from a minority group starts believing that he or she has been treated with discrimination and inequality (Giani and Merlino, 2021). European studies lack research that linksmulticultural education and ethnic discrimination as compared to the studies carried out in the United States. This is, perhaps, for the reason that different focal points are considered in European and American research domains (Agirdag et al., 2016). After discussing the multicultural education system, our attention diverts to its interaction with the composition of ethnic students in educational setups. The ethnic composition of the student population is one of the core variables that have a direct link to ethnic discrimination. Studies highlight that the ratio between the ethnic majority and the ethnic minority has a direct influence on the experience of discrimination. Students' population composition changes the dynamics of experience of discrimination, however, the results from different studies remain inconclusive (Benner and Graham, 2013). It is implied that ethnic composition is a core variable to understand the relationship between the multicultural education system and ethnic discrimination (Lee, 2022). Research suggests that teachers tend to remain more present-minded in a multicultural class where the number of ethnic students is higher as compared to a class where this number is relatively lower (Agirdag et al., 2016). Another study concludes that ethnic composition is an important factor in making a multicultural education system more effective (Thijs and Verkuyten, 2014). For instance, a multicultural message will be well received if the composition of the ethnic student population is higher as the message would seem more relevant.

Social identity theory which is an offshoot of social exchange theory comes is relevant in explaining the causes and consequences of ethnic discrimination. Perceived discrimination is different from actual discrimination indicators because it is not based on objective realities and is rather self-rated (Fischer and Shaw, 1999). Resultantly, it is an indirect measure of a sense of discrimination that is primarily shaped by multiple cognitive factors at play. However, even this subjective account of discrimination is very important in understanding what exactly constitutes a discriminatory act. Perceived discrimination is also important from the research point of view because it provides an opportunity to study and explore the problem without going into an ethical dilemma of exposing someone to discriminatory behavior in a bid to build a dataset (Fischer and Shaw, 1999). There are a lot of consequences for the reaction to an encounter, whether or not that encounter is deemed unfair and/or discriminatory. A situationcan be defined as real in light of the consequences that entail that situation. Therefore, if stress, anxiety and negative impact on the psychological well-being entail even the perceived discrimination that would be considered real keeping in view the consequences (Fleischmann et al., 2019).

The frequency of perceived discrimination is one aspect that can make it stressful. According to a study, there are persistent demands and continuous threats that an individual faces in the wake of perceived discrimination (Williams and Mohammed, 2009). Frequent demands and endless threats result in the exasperation of an individual. There is one perceived measure of ethnic discrimination that occurs frequently which discriminates against a routine or everyday affair for the victim. Frequent discrimination even if it is perceived discrimination is considered a significant and critical source of anxiety and unease (Sanders Thompson, 2006). Even though, the perception of unfair treatment that is subtle but influential causes stress particularly if it occurs at regular intervals or frequently (Lee, 2022). Everyday discrimination is even more stressful given its frequency and can lead to chronic stress. For instance, Essed argues that everyday discrimination based on race can be entirely different from general discrimination based on race because this everyday discrimination is systematic and regular (Essed, 2014).

It is suggested to study these frequent occurrences to dissect how these incidents impact the psychological well-being of victims who have to bear unfair treatment because of their social characteristics (Schulz and Lempert, 2004; Essed, 2014). The extent of the perceived discrimination and the subsequent consequences of unfair treatment of a particular group is an important research area that requires further examination. Racial discrimination is found to be highly detrimental to the emotional well-being of the people at the receiving ends. Such people often feel disempowered and helpless which makes them angry, fearful, and have less control over life events. Discrimination, whether actual or perceived, has detrimental to both physical and mental health (Clark et al., 1999). Minorities and people of the race are often seen reporting mistreatment, unfair attitudes toward them, systematic alienation, and discrimination based on their ethnicity (Williams, 1996).

Innovation has a direct positive link to economic prosperity. Productivity increases, GDP growth sees an upward trend and consumers get to benefit from innovation with the extension of new and improved technologies. Innovation harbors positive change and it extends up to the point where the requirement for its demand is fulfilled (Taques et al., 2021). It is because of these characteristics that many countries while trying to get the journey of their development on the track of innovation tend to consider many different factors and still, sometimes, these efforts do not lead to the desired results (Heinonen and Strandvik, 2021). It is important to note, however, that for innovation to have a positive impact there has to be a low level of resistance to the process of innovation on part of organizations and the economic system as a whole (Rajapathirana and Hui, 2018).

Appropriate conditions are required for a systematic process of innovation to take effect and sustain the organizational pressure that resists it to happen. It is, therefore, highly imperative to understand and recognize the factors and methods that resist innovation, if conditions to create acceptable innovations are to be achieved at all. A particular culture of an organization is highly important for innovational changes. Organizational culture has many facets such as the social and cognitive environment, the collective world-view of reality, beliefs, customs, traditions, and value systems (Jassawalla and Sashittal, 2002). The very initial phases of the process of nelson innovation that require some business-oriented events to happen successfully to make an opening for the change can get negatively hampered by factors and methods of innovation resistance (Ponta et al., 2021). This is one of the core reasons why there is a time lag between discoveries and their streamlining as groundbreaking practical implications. These are forces of tradition in the society that work as resistance to the innovative change in fear of having possible chaotic consequences that an uncertain innovational change can entail (Mokyr, 1998). Therefore, in managing the resistance aspects of innovation attention should not only be given to the availability of energy and technical aspects rather human factors also be focused on equally. In the light of the above literature facts explained under the headings of minority perceived discrimination and resistance to innovation, it can be hypothesized that perceived discrimination has a positive relationship with resistance to innovation.

***H1**: Perceived discrimination has a positive relationship with resistance to innovation*.

### Relationship between perceived discrimination, resistance to innovation, and job dissatisfaction

Diversity has tremendous benefits as well as challenges. However, benefits simply outnumber challenges. An organization that has diversity is better placed to accumulate these benefits. An uptick in productivity, better retention rates, and remains the first and most desired choice of the best talent in the field remain only a few most visible benefits for such organizations (Morrison and Robinson, 1997). This, however, in itself remains one of the biggest challenges for the organizations to first create diversity among their file and ranks and then to maintain it thoroughly. Diversity management is ensured through fair policies and the implementation of appropriate managerial regimes. Another organizational challenge for the maintenance of diversity in a workplace is paying close attention to the perception of employees regarding discrimination (Benner and Graham, 2013). Everyday discrimination blurs the boundaries between institutional discrimination and the perception of an individual toward whom the unfair conduct is directed. For instance, a woman of Afro-American descent may report that she was denied a job role based on her race constitutes an incident of discrimination at one point in time. Though, undoubtedly even this isolated episode will have mental consequences for that particular woman. But the same woman having to face unfair treatment or discrimination during her weekly shopping trips to a local market constitutes a more frequent occurrence of discrimination. This can accumulate over time and has drastic consequences for individuals (Cameron et al., 2020).

Thus, it is hypothesized that perceived discrimination has a positive relationship with job dissatisfaction.

***H2**: Perceived discrimination has a positive relationship with job dissatisfaction*.

The importance of this requirement cannot be exaggerated as the perception of the employees whether or not it tallies with reality is tremendously important because it affects their conduct and behavior at work (Barak et al., 1998). The most important factor for an organization toward their employees remains to have a concrete idea of the level of organizational loyalty and organizational citizenship behavior. Such factors can directly and drastically be affected if the employees have a perception that they remain victims of discrimination for whatsoever reason. For productivity, job satisfaction is the primary requirement, and job satisfaction comes from the sense that one is being taken care of appropriately and based on equality. Perceived discrimination greatly impedes job satisfaction (Locke, 1976). There has been a lot of work carried out to understand different facets and aspects of job satisfaction, nevertheless, Jayaratne found that racial discrimination as an aspect that affects the level of job satisfaction has not been studied in depth (Jayaratne, 1993). This is perhaps the reason why a scientific consensus has not reached as far as the role of variables such as racial discrimination, perceived discrimination, and job satisfaction (Cox and Nkomo, 1993).

The effect of perceived discrimination among Hispanic employees was studied to know its impact on job outcomes (Sanchez and Brock, 1996). The study found that they experienced higher work-related tension and were less satisfied with their job in the wake of perceived discrimination. Their organizational commitment was not as high as others. Furthermore, the study interestingly found that well-placed employees in sense of position or salary did not face as much discrimination as those who were low wages or held positions of less influence. From the above discussion, it is hypothesized that perceived discrimination has a positive relationship with job dissatisfaction, which in turn has a positive relationship with resistance to innovation. It is also hypothesized that job dissatisfaction mediates the relationship between perceived discrimination and resistance to innovation.

***H3**: Job dissatisfaction has a positive relationship with resistance to innovation*.

***H4**: Job dissatisfaction mediates the positive relationship between perceived discrimination and resistance to innovation*.

### Psychological distress as a moderator between perceived discrimination, resistance to innovation, and job dissatisfaction

Psychological distress is defined as a combination of non-specific symptoms of depression, anxiety, and stress which indicate mental disorders and impaired psychological health. Psychological distress is caused by multiple factors including various social, economic, biological, and physical factors. Various societal roles, workplace roles and responsibilities, and institutional responsibilities can lead to psychological distress among individuals (Keles et al., 2020). Studies have shown an increased level of psychological distress among students (Li et al., 2021). Health workers and medical students show raised levels of psychological distress with impaired mental and physical health (Lorente et al., 2021). More and more universities and academic institutions have started to recognize psychological distress as a risk to the mental well-being of learners. Royal College of Psychiatrists' concern regarding the mental health of the students after their initial probe into the question of mental health of the students. And recently, they have highlighted that matter has become even more grave. Similarly, the National Survey of Counseling Center Directors that was carried out in 2002 found that 83% of the students were struggling with severe psychological problems (Gallagher, 2010). Now, after more than a decade the situation has gone from bad to worse as the percentage of students having compromised psychological health soared past 93.7% (Gallagher, 2013). These concerns highlighted by the field practitioners have been validated through empirical research. Psychological distress has not only limited to a certain kind of students rather it remains a phenomenon true for all kinds of students globally. University students have been provided prevalence over the general population as far as psychological distress is concerned. There are much broader implications of elevated psychological distress among university students. They tend to have poor mental as well as physical health along with impaired academic performance. Scientific literature identifies key factors responsible for giving rise to psychological distress and intervention points have also been identified to help those struggling with psychological distress. The role of perceived discrimination as a factor giving rise to stress has increasingly been recognized to help understand health disparities (Jackson et al., 1995; Kessler et al., 1999; Schulz et al., 2000; Taylor and Turner, 2002; Williams and Mohammed, 2009). Different aspects such as perceived discrimination in a group is now increasingly being recognized to better study the complex processes that impact mental health (Smith et al., 1992; Essed, 2014). The impact of everyday or recurrent discrimination depends on the availability or presence of resources that are required to ameliorate the negative effect of everyday discrimination. Therefore, the current study hypothesizes that psychological distress moderates the positive relationship between perceived discrimination and resistance to innovation.

***H5**: Psychological distress moderates the positive relationship between perceived discrimination and resistance to innovation*.

Job dissatisfaction is a situation when someone dislikes or is not satisfied and content with his or her work life. Studies have revealed the negative effects of job dissatisfaction on the mental health and social life of individuals. It has immensely negative consequences for both physical and psychological health (Extremera et al., 2020). According to a study, job dissatisfaction affects learning motivation, and creativity among students (Wijaya, 2019). Job dissatisfaction causes dropouts from workplaces. According to a previous study job dissatisfaction act as a mediator to enhance the intention of leaving a job due to job dissatisfaction (Chen et al., 2019). Workers' well-being is often measured through the level of their job dissatisfaction since it incorporates micro- as well as macro-level interaction of an employee at his/her workplace. Job dissatisfaction is linked with psychological problems that include exhaustion, burnout, stress, anxiety, and lack of self-esteem (Fitzgerald et al., 2003). The same was found in a metanalysis that was based on almost 500 previous studies that explored the factors that lead to job dissatisfaction and also the consequences for the physical and mental health of the employees who experienced job dissatisfaction. The literature highlights that job dissatisfaction is one of the core predictors of health indicators (Billah et al., 2021). The research hypothesizes that job dissatisfaction has a direct relation to distress and physical health issues. The research considered many diverse factors to better understand the relationship. From the inception of scientific ideas to their practical commercialization, the process of innovation is complex. Innovation is a stochastic process as its pattern is random and uncertain. Though it may be possible to analyze it statistically, however, it remains next to impossible to predict precisely (Pinder, 2014). Resistance to innovation arises when the feedback in the economic system is not positive. Some social groups and sub-systems resist the incorporation of innovation for a variety of reasons. The process of adoption of the innovation by social groups is languished and may take a while. Negative psychological and social perceptions slow down the process of innovation. Though individuals, social groups, and organizations may not have the capacity and influence to stop innovation from taking effect, however, they do have this tendency and power to at least erect considerable bottlenecks to keep the process moving slowly (Inuwa, 2016).

Keeping in view the above-mentioned facts from literature, it is hypothesized that psychological distress moderates the positive relationship between job dissatisfaction and resistance to innovation.

***H6**: Psychological distress moderates the positive relationship between job dissatisfaction and resistance to innovation*.

**Figure 1:**
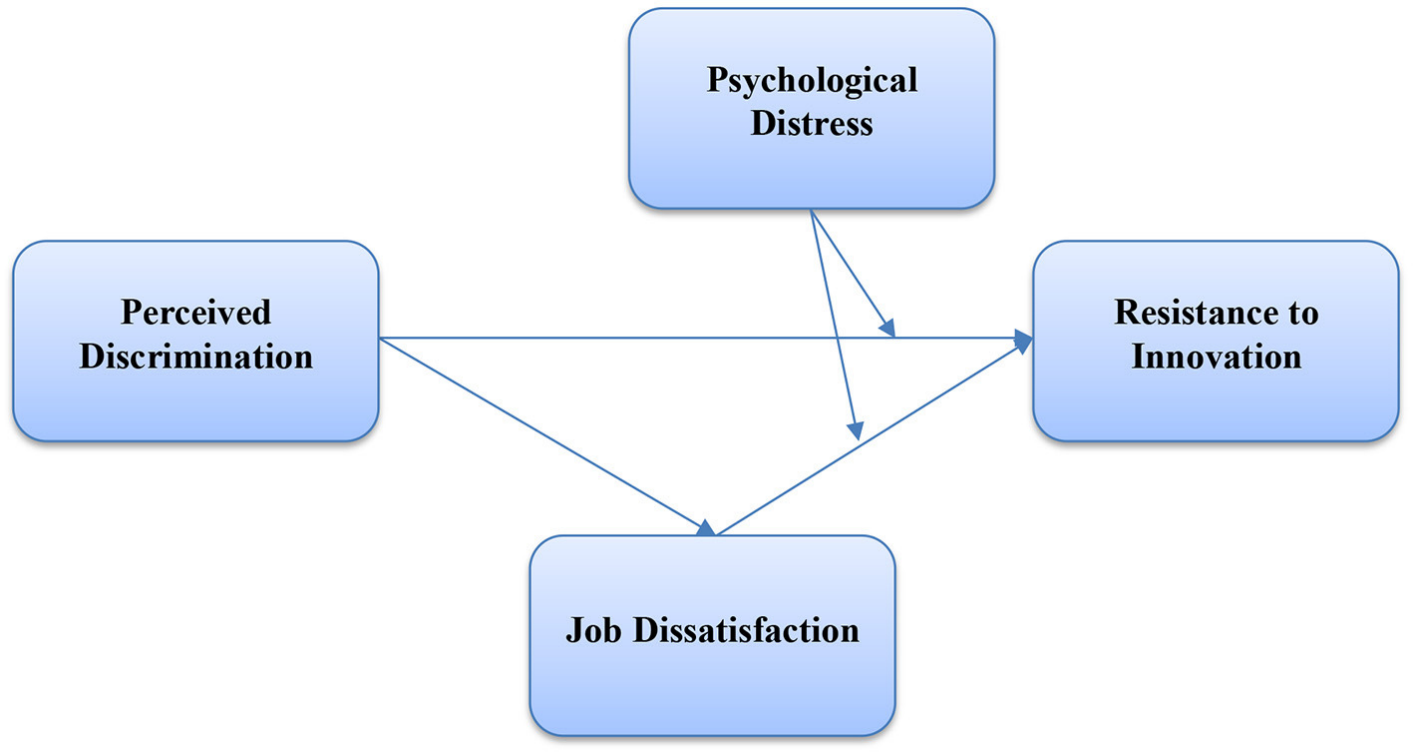
Conceptual framework.

The present study's conceptual framework is given in Figure 1.

## Research methods

### Study design

This study was conducted with the objective of examining the role of minority perceived discrimination on their behavior of resistance to innovation. According to our research objective, this study adopted a purposive sampling technique to target specific people, such as ethnic minorities in China (Taherdoost, 2016). These ethnic minority students were students of MBA executives as they were also doing jobs in the morning and their studies in the evening. A meeting was conducted to guide the authorities regarding the data collection objective for getting their permission and corporation in this job. As the present study needed the details of the minority student, two further meetings were organized to convince them regarding the permission for data collection by ensuring their data confidentiality. It was revealed that data would be used for academic aims, and the outcomes would also be shared with them in the shape of practical implications. Finally, the heads of universities permitted data collection and agreed to share the details of their minority students.

To not influence and confidence the minority students, it was decided to develop the questionnaires in google forms to get online responses. Before data distribution, an online meeting was also conducted with them. For an online meeting, a formal request was made through a letter explaining the objective and importance of this study. It was also assured that privacy is the main priority, such as ensuring their data's strict privacy. A WeChat group was shared with them to join for questionnaire filling. The right or wrong answers scenario was explained to get natural responses from them, such as all their true answers would be considered appropriate for this study. This way, their confidence was boosted, and they enthusiastically participated in this study. Moreover, for the convenience of the students, the questionnaires were translated into Chinese under the senior researchers' guidance, and the senior researchers also approved the final questionnaire after getting some sample-based data and correcting some language errors. This way, dual language questionnaires were developed to collect the data.

The questionnaires were distributed in the WeChat group. A total of 339 students joined the group. The present study took 1 month to collect the data from the students. The author weekly pushed the students in the group with soft and polite reminders. In the first week, a total of 135 responses were received. In the second week, 110 responses were received from the students. Similarly, in the third week, the present study collected 69 responses, and in the last week, 14 more responses were received back, ending the data collection process. This way, this study received 328 complete and valid responses for the empirical analyses of the present study. The outcomes of the present study depend on the 328 sample size appropriate for structural equation modeling (Rahi, 2017).

### Measures

This study used five points Likert scale to measure the participants' responses. This scale consists of five numbers where 1 means “strongly disagree,” 2 means “disagree,” 3 means “neutral,” 4 means “agree,” and 5 means “strongly agree.” This study considered previously validated items to assess the variables.

#### Perceived discrimination

The construct perceived discrimination measurement scale was adapted from Sanchez and Brock (1996). This scale has ten items. The sample item is “At work, I feel uncomfortable when others make jokes or negative commentaries about people of my ethnic background.” The Cronbach alpha value is 0.920.

#### Job dissatisfaction

The construct of job dissatisfaction was measured with a six-item scale used by De Clercq et al. (2019). The sample item included, “I am often bored with my job.” The Cronbach alpha valued is 0.879.

#### Resistance to innovation

Resistance to innovation was measured using a five-item scale adapted from Mani and Chouk (2018). The sample item included, “Sample item included, ”The use of new technologies would be connected with too many uncertainties.“ The Cronbach alpha value is 0.875.

#### Psychological distress

Psychological distress was measured with three items scale developed by Barnett and Brennan (1995) and used by Lapalme et al. (2009). The sample item included, ”I feel worried and anxious.“ The Cronbach alpha value is 0.820.

## Results

### Assessment of measurement and structural model

**Figure 2:**
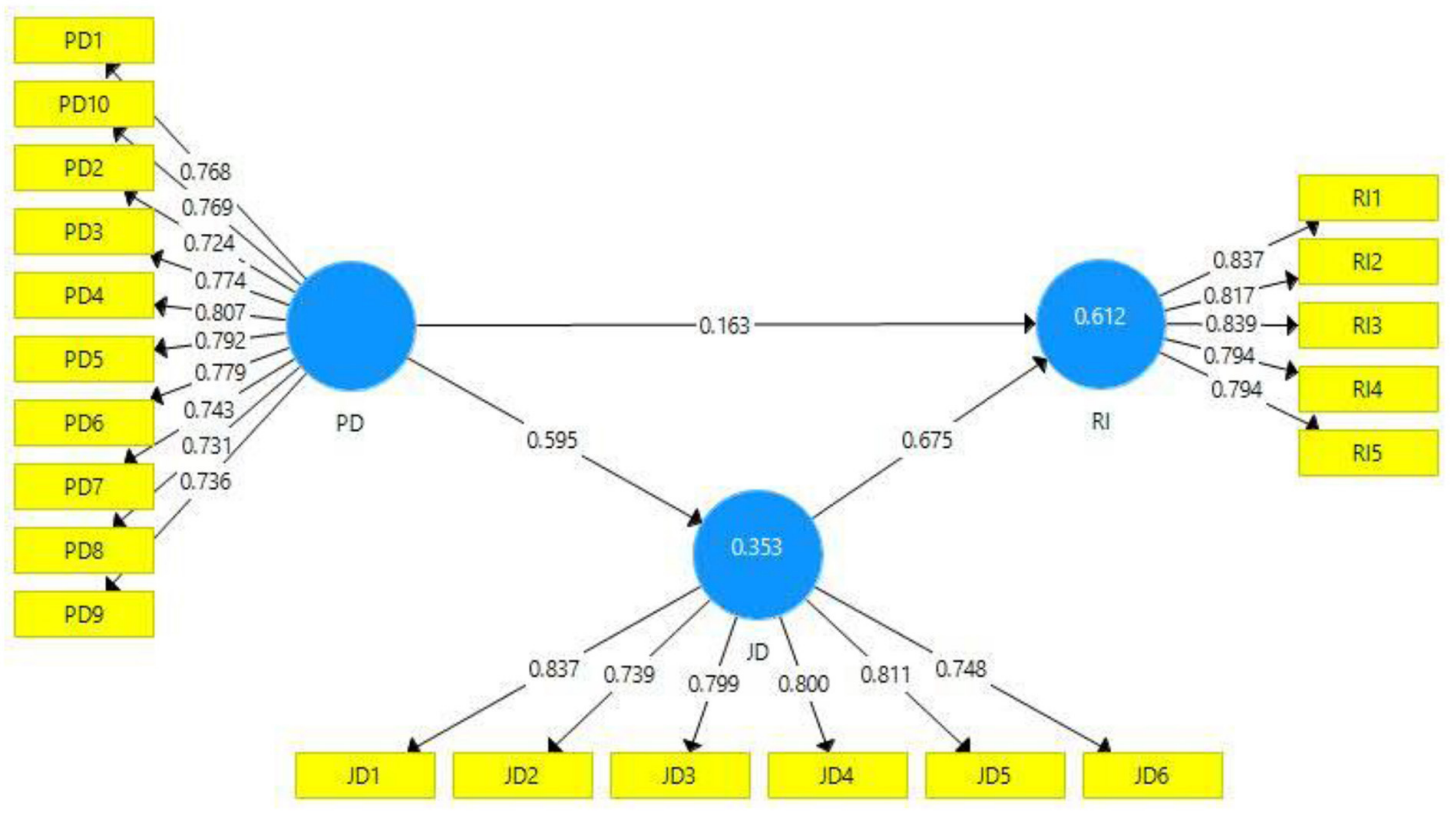
Path estimates and outer loadings (Mediation).

CB-SEM and PLS-SEM are two different methods of structural equation modeling (SEM) (Hair et al., 2019a). The primary distinction between the two methodologies is that PLS-SEM is used to advance and build theories, whilst CB-SEM is used to evaluate and accept hypotheses (Bashir et al., 2021). PLS-SEM is a suitable method for complicated and multi-order models with no explicit requirements for data normality. Small data sets can also be evaluated efficiently using PLS-SEM (Bari et al., 2019). This present study considers each construct to evaluate the reliability of the model. The outer loading values are considered reliable if they exceed 0.70 (Hair et al., 2014). Table 1 depicts that all outer loading values of the present study's constructs are according to acceptable criteria (Figure 2). Further, this study assessed the values of “Cronbach's alpha, composite reliability, and average variance extract (AVE)” to validate the model's reliability. According to Hair Jr et al. (2016), the acceptance criteria for the values of Cronbach's alpha and composite reliability is 0.70. Table 1 shows that the Cronbach's alpha values are (job dissatisfaction, perceived discrimination, and resistance to innovation) 0.879, 0.920, 0.875, and the values for composite reliability are 0.908, 0.933, and 0.909, respectively. The values of Cronbach's alpha and composite reliability confirmed the model's reliability. The AVE values are used to verify the convergent validity of the construct, and the values are accepted if they are >0.5 (Hair et al., 2019b). The AVE values of the current study are presented in Table 1. The AVE values are (0.623, 0.582, and 0.667) according to the required standards.

**Table 1:** Reliability and convergent validity of the study constructs (Mediation).

**Construct**	**Item**	**Outer loadings**	**VIF**	**Alpha**	**roh-A**	**Composite reliability**	**AVE**
JD	JD1	0.837	2.414	0.879	0.881	0.908	0.623
	JD2	0.739	1.869				
	JD3	0.799	1.995				
	JD4	0.800	2.008				
	JD5	0.811	2.071				
	JD6	0.748	1.640				
PD	PD1	0.768	2.263	0.920	0.922	0.933	0.582
	PD2	0.724	1.952				
	PD3	0.774	2.429				
	PD4	0.807	3.077				
	PD5	0.792	2.591				
	PD6	0.779	2.267				
	PD7	0.743	2.260				
	PD8	0.731	2.395				
	PD9	0.736	2.100				
	PD10	0.769	2.291				
RI	RI1	0.837	2.164	0.875	0.877	0.909	0.667
	RI2	0.817	2.012				
	RI3	0.839	2.226				
	RI4	0.794	1.981				
	RI5	0.794	1.978				

JD, Job Dissatisfaction; KH, Perceived Discrimination; RI, Resistance to Innovation.

For assessing the discriminant validity of the current study, two important approaches are used, including the “Fornell–Larcker criterion and heterotrait–monotrait (HTMT) ratio” (Hair et al., 2019a). The Fornell–Larcker criterion values could be assessed by having the square root of AVE values. According to the required criteria, these square root values must exceed the other variables' correlation values in a similar column (Hair Jr et al., 2016). The discriminant validity would be considered ideal if the values of constructs are <0.85; however, the values <0.90 could also be considerable. Tables 2, 3 acknowledge that the values of Fornell–Larcker criterion and HTMT ratios meet the required criteria; hence it is a positive indicator for the formation of the discriminant validity.

**Table 2:** Discriminant validity (Fornell-Larker-1981 Criteria) (Mediation).

**Construct**	**JD**	**PD**	**RI**
**JD**	**0.790**		
**PD**	0.595	**0.763**	
**RI**	0.771	0.564	**0.816**

JD, Job Dissatisfaction; KH, Perceived Discrimination; RI, Resistance to Innovation. The bold values indicate the results for corresponding statistics for whole variable not the items.

**Table 3:** Discriminant validity (HTMT) (Mediation).

**Construct**	**JD**	**PD**	**RI**
**JD**	–	–	–
**PD**	0.656	–	–
**RI**	0.872	0.626	–

JD, Job Dissatisfaction; KH, Perceived Discrimination; RI, Resistance to Innovation.

The VIF values are assessed to validate the collinearity issues in the model. The VIF values <0.5 are considered ideal and indicate that the model is free from collinearity issues (Hair et al., 2011). According to the findings in Table 1, all VIF values are lower than 0.5, such as the variable “perceived discrimination” item PD-4has the highest VIF value (3.077). Hence, it is verified that there are no collinearity concerns in the model of the current study. The current study also confirmed that *R*^2^ and *Q*^2^ values of all endogenous constructs of this study (job dissatisfaction and resistance to innovation) show moderate model strength.

### Model estimation direct and indirect relationship

For model evaluation, this study applied a bootstrapping technique with 5,000 random samples replacement. The direct, indirect, and total paths (mediation analysis) are presented in Table 4. This study assessed the “*t*” and “*p*” statistics values as a criterion for the acceptance or rejection of the hypotheses. The current study hypothesis results are shown in Table 5. According to hypothesis 1, perceived discrimination has a positive relationship with resistance to innovation, and the outcomes (*t* = 3.158, *p* = 0.002) confirmed that H1 of the present study is accepted. The beta value of H1 acknowledged that one-unit variation in the independent variable (perceived discrimination) would result in0.163changes in the dependent variable (resistance to innovation). The outcomes (*t* = 7.786, *p* = 0.000) of the second hypothesis confirmed that perceived discrimination has a positive relationship with job dissatisfaction, and it is verified that H2 of the present study is accepted. The beta value shows that one unit change in perceived discrimination would result in 0.595 changes in job dissatisfaction. According to the results (*t* = 13.665, *p* = 0.000) of the third hypothesis, job dissatisfaction has a positive relationship with resistance to innovation. Hence, it is confirmed that H3 of the present study is accepted. The beta value shows that one unit change in job dissatisfaction would cause the 0.675 changes in resistance to innovation.

**Table 4:** Direct, Indirect and Total path estimates (Mediation).

**Direct path**	**Beta**	**SD**	**t**	**p**
JD -> RI	0.675	0.049	13.665	0.000
PD ->JD	0.595	0.076	7.786	0.000
PD -> RI	0.163	0.052	3.158	0.002
**Indirect path**				
PD -> JD -> RI	0.401	0.070	5.696	0.000
**Total path**				
JD -> RI	0.675	0.049	13.665	0.000
PD ->JD	0.595	0.076	7.786	0.000
PD -> RI	0.564	0.081	6.991	0.000

JD, Job Dissatisfaction; KH, Perceived Discrimination; RI, Resistance to Innovation.

**Table 5:** Hypotheses testing (Mediation).

**Hypotheses**		**Coefficient (Beta)**	**S.D**	***t***	***p***	**Status**
H1	PD -> RI	0.163	0.052	3.158	0.002	Supported
H2	PD -> JD	0.595	0.076	7.786	0.000	Supported
H3	JD -> RI	0.675	0.049	13.665	0.000	Supported
**Mediation hypotheses**						
H4	PD -> JD -> RI	0.401	0.070	5.696	0.000	Supported

JD, Job Dissatisfaction; KH, Perceived Discrimination; RI, Resistance to Innovation.

This study also considered the mediating role of job dissatisfaction mediates the relationship between perceived discrimination and resistance to innovation. For the empirical examination of job dissatisfaction as a mediator, this study H4. Results of H4 (*t* = 5.696, *p* = 0.003) confirm thatjob dissatisfaction mediates the relationship between perceived discrimination and resistance to innovation. The path value (0.401) also confirms that H4 of this study is accepted.

### Moderation analysis

The present study considers a two-stage method for moderation analysis, including model measurement and model estimation. The moderation analysis of the present study elucidated that all basic criteria of constructs' reliability and validity are achieved. The indicators of model assessment, such as “outer loading values, CR, Cronbach's alpha, and AVE”, are also according to acceptable criteria (Hair et al., 2019b). Table 6 presents the details of model assessment indicators.

**Table 6:** Reliability and Convergent Validity of the Study Constructs (Moderation).

**Construct**	**Item**	**Outer loadings**	**VIF**	**Alpha**	**roh-A**	**Composite Reliability**	**AVE**
JD	JD1	0.837	2.414	0.879	0.881	0.908	0.623
	JD2	0.739	1.869				
	JD3	0.799	1.995				
	JD4	0.800	2.008				
	JD5	0.811	2.071				
	JD6	0.748	1.640				
PD	PD1	0.768	2.263	0.920	0.922	0.933	0.582
	PD2	0.724	1.952				
	PD3	0.774	2.429				
	PD4	0.807	3.077				
	PD5	0.792	2.591				
	PD6	0.779	2.267				
	PD7	0.743	2.260				
	PD8	0.731	2.395				
	PD9	0.736	2.100				
	PD10	0.769	2.291				
PsYD	PsYD1	0.808	1.584	0.820	0.823	0.893	0.736
	PsYD2	0.897	2.330				
	PsYD3	0.868	2.041				
RI	RI1	0.837	2.164	0.875	0.876	0.909	0.667
	RI2	0.817	2.012				
	RI3	0.839	2.226				
	RI4	0.794	1.981				
	RI5	0.794	1.978				

JD, Job Dissatisfaction; KH, Perceived Discrimination; RI, Resistance to Innovation; PsYD, Psychological Distress.

The outcomes of moderation analysis verified the discriminant validity with moderation effect (psychological distress) by using two approaches, “Fornell–Larcker criterion and HTMT ratios.” Tables 7, 8 describe the findings of the Fornell–Larcker criterion and HTMT ratios. The results also illuminated that the inner VIF values of all constructs are significantly lower than 5 (Table 5), whichindicates that the study is free fromthe collinearity issue. The R^2^ values of endogenous variables of the current study's model show moderate model strength, which is a good indicator of model significance (Hair et al., 2012).

**Table 7:** Discriminant Validity (Fornell-Larker-1981 Criteria) (Moderation).

**Construct**	**JD**	**PD**	**PsYD**	PsYD*JD	PsYS*PD	**RI**
**JD**	**0.790**					
**PD**	0.595	**0.763**				
**PsYD**	−0.651	−0.575	**0.858**			
PsYD*JD	0.714	0.614	−0.556	**1.000**		
PsYS*PD	0.701	0.586	−0.601	0.949	**1.000**	
**RI**	0.770	0.565	−0.749	0.705	0.691	**0.816**

JD, Job Dissatisfaction; KH, Perceived Discrimination; RI, Resistance to Innovation; PsYD, Psychological Distress.

**Table 8:** Discriminant validity (HTMT) (Moderation).

**Construct**	**JD**	**PD**	**PsYD**	PsYD*JD	PsYS*PD	**RI**
**JD**	–	–	–	–	–	–
**PD**	0.656	–	–	–	–	–
**PsYD**	0.758	0.651	–	–	–	–
PsYD*JD	0.761	0.640	0.615	–	–	–
PsYS*PD	0.746	0.607	0.665	0.949	–	–
**RI**	0.872	0.626	0.883	0.754	0.738	–

JD, Job Dissatisfaction; KH, Perceived Discrimination; RI, Resistance to Innovation; PsYD, Psychological Distress.

### Model estimation moderation

The present study also assumed the moderating role of psychological distress in the relationship between perceived discrimination and resistance to innovation, and the relationship between job dissatisfaction and resistance to innovation, respectively. For empirical investigation present study assumes H5 and H6. The results (*t* = 1.463, *p* = 0.144) revealed that psychological distress does not moderate the relationship between perceived discrimination and resistance to innovation; therefore, H5 of the current study is not accepted (Table 9). Additionally, according to the results of the sixth hypothesis (*t* = 3.537, *p* = 0.000),psychological distress moderates the relationship between job dissatisfaction and resistance to innovation.

**Table 9:** Hypotheses Testing (Moderation).

	**Moderation Hypotheses**	**Coefficient (Beta)**	**S.D**	***t***	***p***	**Status**
H5	PsYD*PD -> RI	−0.074	0.050	1.463	0.144	Not Supported
H6	PsYD*JD -> RI	0.151	0.043	3.537	0.000	Supported

JD, Job Dissatisfaction; KH, Perceived Discrimination; RI, Resistance to Innovation; PsYD, Psychological Distress.

**Figure 3:**
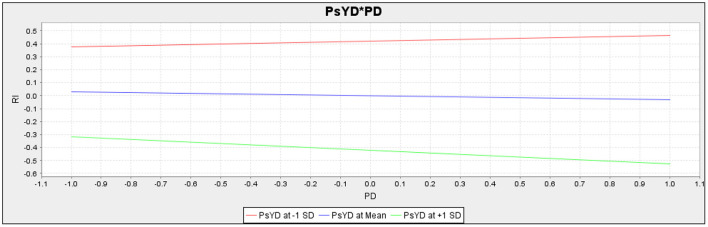
Slope for Psychological Distress (PsYD) and Perceived Discrimination (PD) (Moderation).

**Figure 4:**
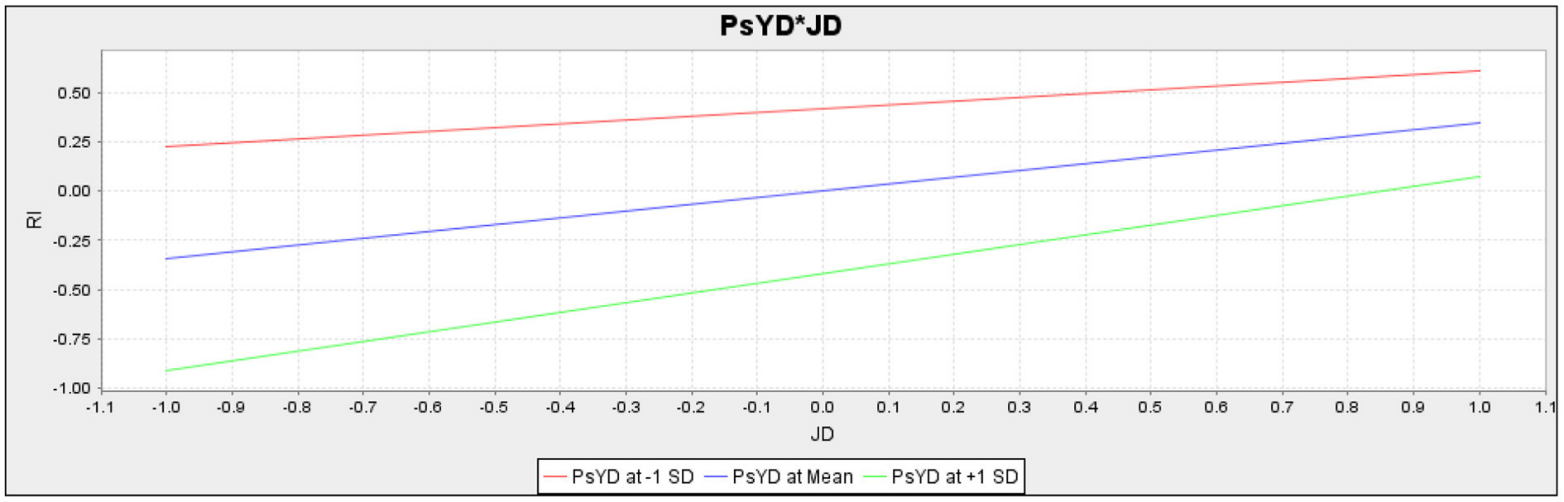
Slope for Psychological Distress (PsYD) and Job Dissatisfaction (JD) (Moderation).

Psychological distress does not moderate the slope for the association between perceived discrimination and resistance to innovation. The slope for the moderation analysis of H5 is given in Figure 3. However, Figure 4 depicts that psychological distress moderates the slope for the association between job dissatisfaction and resistance to innovation.

## Discussion

According to scholars, ethnic discrimination is one of the crucial social issues that need researchers' attention (Verkuyten, 1998; Drejeris and Drejeriene, 2021; Andrade, 2022). Further, prejudice and stereotypescould be possible forms of discrimination that ethnic groups face. Consequently, these groups are underprivileged socioeconomically and educationally. According to Liebkind et al. (2004) arguments, when other groups show negligence toward ethnic groups, they may develop negative feelings and perceptions about them. The psychological and emotional health of group members is influenced when they face discriminant behavior from others (MacGregor et al., 2020). It is also noticed that the well-being and self-esteem of group members are negatively impacted when ethnic group members perceive that other people show discriminant behavior toward them. Consequently, minority groups feel a sense of inferiority complex that can directly influence their personal and professional life. A prior study revealed that ethnic group members' perception of discrimination adversely influences their attitude and behavior, decreasing their work effectiveness and job performance (Hanassab, 2006).

When ethnic groups perceive themselves as victims ofdiscrimination, they may develop negative behavior about their workplace. The perceived discrimination unfavorably influences their innovation adoption behavior, and in return, they may show resistance to innovation. Based on the social exchange theory, the present study attempts to check the impact of perceived discrimination on resistance to the innovation of ethnic groups. For empirical investigation, this study proposes that perceived discrimination positively correlates with resistance to innovation and job dissatisfaction, respectively. This study further hypothesized that job dissatisfaction is positively associated with resistance to innovation. The current study also assumes the mediating role of job dissatisfaction in the relationship between perceived discrimination and resistance to innovation. Moreover, it is also hypothesized that psychological distress moderates the relationship between perceived discrimination and resistance to innovation and the association between job dissatisfaction and resistance to innovation, respectively.

The results of the current study showed that perceived discrimination positively influences resistance to innovation, which confirmed that the first hypothesis of this study is accepted. When ethnic groups believe they are the targets of prejudice, they may behave badly at work. Their behavior in adopting innovations is negatively impacted by the perceived prejudice, and as a result, they opposed innovation. MacGregor et al. (2020) noticed that perceived discrimination damages the health of individuals and causes a severe type of chronic stress. When other members of an institution show discriminant behavior toward ethnic group members, it causes biases and negatively impacts interpersonal relationships in the workforce (Cronin et al., 2012). Further, a prior study noticed that this situation would be worse in an educational setting where the perceived discrimination of ethnic minority students could cause devastating consequences for their academic careers (Hanassab, 2006). Moreover, the perception of students that their professors discriminate against them on their ethnic basis is a demanding situation for the critical abilities and academic efficacy of students. The discriminant behavior of teachers also affects the mental and emotional health of students, which is also an adverse indicator of students' academic performance. Verkuyten (1998) noticed that the perceived discrimination of ethnic groups is also damaging their self-esteem, and they may develop counterproductive work behavior for institutions, and in return, they may not want to participate in the innovational activities of firms. Additionally, this worse situation causes to develop resistance to innovative behavior in ethnic group members.

The results of the current study further revealed that perceived discrimination positively influences job dissatisfaction, which confirmed that the second hypothesis is also accepted. People feel dissatisfied and frustrated when they perceive that they are experiencing discrimination by others in the workplace. Andrade (2022) also noticed that the job satisfaction of ethnic group members decreases when they perceive that they are facing discrimination from others. Further, it is also observed that the perception of discrimination has devastating consequences on the mental health and well-being of ethnic group members. Additionally, when ethnic group members face discriminant behavior from others, their psychological contract breaches, and in return, their job satisfaction and work productivity decrease adversely (DelCampo et al., 2010; Heads et al., 2021). The results of this study further revealed that job dissatisfaction has a positive relationship with resistance to innovation, which confirmed that H3 is accepted. Drosos et al. (2021) noticed that individuals' satisfaction is an important indicator of acceptance of innovation. Further, they observed that job dissatisfaction leads individuals to resist change and innovation and could also pave the way for counterproductive work behavior.

The outcomes further elucidated that H4 of the present study is accepted, which confirmed that job dissatisfaction positively mediates the association between perceived discrimination and resistance to innovation. The results further revealed that psychological distress does not moderate the correlation between perceived discrimination and resistance to innovation. Hence, the fifth hypothesis is rejected. The reason behind this rejection is that the emotional intelligence of respondents is high and they have the ability to efficiently manage their stress levels. Wang and Zhang (2020) also noticed that emotional intelligence might favorably assist people in coping with adverse environmental strain. High emotional intelligence people frequently experience less strain and are more likely to improve their mental health. Additionally, Fellner et al. (2012) also argue that emotional intelligence can help safeguard the development of one's mental health. On the other hand, the results confirmed that psychological distress moderates the relationship between job dissatisfaction and resistance to innovation, which confirmed that H6 of this study is accepted. Andrade (2022) acknowledged that when ethnic group members face discrimination behavior by other groups, their mental health, and psychological well-being are adversely impacted, lowering their work performance and efficacy. It is also observed that these individuals may suffer from mental health disorders like stress and anxiety.

## Implications

### Theoretical implications

The current research study has multiple theoretical and practical implications which makes the study practical and significant. The core objective of the study was to explore and demonstrate the impact of minority perceived discrimination on resistance to innovation while studying the moderating role of psychological distress. The current study has contributed to the existing literature on social exchange theory by using a unique framework. Furthermore, this article has contributed significantly by adding literature on the mediating role of job dissatisfaction and moderating role of psychological distress. The study demonstrates that perceived discrimination has a positive relationship with resistance to innovation and job dissatisfaction. Furthermore, it has been explored in this study that job dissatisfaction has a positive relationship with resistance to innovation.

The uniqueness of this research study has increased its applications and implications.

It provides literature on the resistance to innovation, job dissatisfaction, and minority perceived discrimination in a single article. It is important to mention that no research has been conducted on the given subject using these variables. This research study provides valuable theoretical literature on the said subject which can be referred to by the researchers, academicians, educationists, and teachers for carrying out further research, expanding the available literature, and designing new research studies.

### Practical implications

In any organization, innovation is the key to survival and competence. If the members of the organization are experiencing any psychological issues like distress which leads to negative outcomes like resistance to innovation and job dissatisfaction then the organization can face failures. Thus, the results of this study should be consulted by the managers, and policy makers to avoid such failures. The ethnic members of any organization should be provided with equal rights to promote a homogenous and employee-friendly environment. It is crucial to study the factors like psychological distress and job dissatisfaction to include minorities and ethnic students and employees in any organization and to provide these people with equal representation and opportunities. Mental health has been a matter of great concern in present times. Therefore, any factor affecting the mental health of students and employees of any organization needs to be studied for better policy making. The study has explored the mediating role of psychological distress and job dissatisfaction. This mediating role must be understood by all the stakeholders to raise the level of job satisfaction, innovation, and self-esteem in the ethnic members of the organization. The results of this study can be used practically to enhance the performance of the ethnic students which will ultimately increase the positive outcomes of the organization.

## Limitations

The present study serves the literature by providing insights into different aspects of the consequences of perceived discrimination against ethnic groups. However, this study also has some limitations, which may become opportunities for scholars in the future. First, this study attempts to check the role of perceived discrimination in resistance to the innovation of ethnic groups. Future studies may try to reveal the impacts of perceived discrimination on the psychological and emotional health of ethnic groups. Future studies may also consider the impact of perceived discrimination on the critical abilities and work productivity of ethnic group members. Second, this study assumes the mediating role of job dissatisfaction in the relationship between perceived discrimination and resistance to innovation. Future studies may consider other mediating variables such as cynicism and psychological contract breach, etc. Third, the current study assessed psychological distress as a moderating variable; however, future studies may consider other moderating variables to extend the finding of this study, like counterproductive work behavior, etc. Fourth, this study collected data through a structured questionnaire; future studies may adopt other data collection methods such as semi-structured questionnaires or interview methods. Fifth, this study is conducted with a small sample size; future studies may extend the sample size to validate the results of this study. Sixth, the current study is conducted in China, and the findings may not be generalizable to other contexts. In the future, this study may be conducted in other developing or developed countries to better understand the study's objectives.

## Conclusion

Ethnic discrimination is one of the crucial social issues that need researchers' attention. Ethnic minority groups usually face discrimination in the form of prejudice and stereotypes. The discriminant behavior of others adversely impacts the psychological and emotional health of ethnic minority groups. The discriminant behavior of others negatively influences the well-being of these individuals. Perceived discrimination has created a sense of inferiority complex in ethnic minority groups. When ethnic groups perceive discrimination, their work productivity and performance are negatively influenced. The critical abilities and innovation adoption also decline badly when ethnic groups perceive themselves as victims ofdiscrimination. Based on the social exchange theory, this study aims to determine the impact of perceived discrimination on resistance to the innovation of ethnic groups. For empirical investigation, this study proposes six hypotheses. According to the first andsecond hypotheses, perceived discrimination positively correlates with resistance to innovation and job dissatisfaction, respectively. Third,job dissatisfaction has a positive relationship with resistance to innovation. Fourth, this study also assessed the mediating role of job dissatisfaction between the association of perceived discrimination and resistance to innovation. According to the fifth and sixth hypotheses, psychological distress moderates the correlationbetween perceived discrimination and resistance to innovation and the relationship between job dissatisfaction and resistance to innovation. The present study confirmed that perceived discrimination positively impacts resistance to innovation and job dissatisfaction, respectively. The results further verified that job dissatisfaction positively influenced resistance to innovation. The current study's findings further authenticated that job dissatisfaction positively mediates the association between perceived discrimination and resistance to innovation. Moreover, the outcomes revealed that psychological distress does not moderate the relationship between perceived discrimination and resistance to innovation. On the other hand, the study's results also confirmed that psychological distress moderates the relationship between job dissatisfaction and resistance to innovation. The current study serves the literature by providing empirical evidence on the ethnic minority groups' perception of discrimination. Moreover, this study shed light on the possible consequences of the perception of discrimination. This study revealed that perceived discrimination decreases job satisfaction and enhances the resistance to innovation in ethnic groups.

## Data availability statement

The original contributions presented in the study are included in the article/supplementary material, further inquiries can be directed to the corresponding author/s.

## Ethics statement

The studies involving human participants were reviewed and approved by School of Marxism, Shandong Yingcai University, China. The patients/participants provided their written informed consent to participate in this study. The study was conducted in accordance with the Declaration of Helsinki.

## Author contributions

JW: conceptualization and data collection. MS: writing the draft. JW and MS: agreed to the submitted version of manuscript. Both authors contributed to the article and approved the submitted version.

## Conflict of interest

The authors declare that the research was conducted in the absence of any commercial or financial relationships that could be construed as a potential conflict of interest.

## Publisher's note

All claims expressed in this article are solely those of the authors and do not necessarily represent those of their affiliated organizations, or those of the publisher, the editors and the reviewers. Any product that may be evaluated in this article, or claim that may be made by its manufacturer, is not guaranteed or endorsed by the publisher.
